# Analysis and mapping of global research publications on shift work (2012–2021)

**DOI:** 10.1186/s12995-022-00364-0

**Published:** 2022-12-14

**Authors:** Waleed M. Sweileh

**Affiliations:** Department of Physiology, Pharmacology/Toxicology, Division of Biomedical Sciences, College of Medicine and Health Sciences, An-Najah National University, Nablus, Palestine

**Keywords:** Shift work, Research publications, Bibliometric analysis, Visualization maps

## Abstract

**Background:**

The main objective of the study was to identify research trends, collaboration patterns, and the most impactful publications in the field of shift work.

**Methods:**

Documents published in scientific journals indexed in the Scopus database on shift work were retrieved and analyzed using the title/abstract search methodology. The study period was from January 1st, 2012, to December 31st, 2021.

**Results:**

Two thousand three hundred twenty-eight documents were retrieved with an h-index of 71 and an average number of 4.5 authors per document. The cumulative number of publications showed a linear growth pattern, while that of citations showed an exponential pattern. The most frequent author keywords, excluding keywords related to shift work, were sleep, fatigue, and nursing. The average annual growth rate was 4.3, and the average doubling time was 3.2. No significant correlation was found between the number of publications and national income among prolific countries. Cross-country research collaboration was weak while the degree of author-author collaboration was relatively high. The *Chronobiology International* journal was the most prolific, while *Harvard University* was the most active institution in the field of shift work.

**Conclusions:**

Given the volume and the negative health impact of shift work, better human resource management is needed to create safer and healthier working schedules.

## Background

Shift work is defined as working, either permanently or periodically, at times that are outside the standard working hours (7 or 8 a.m. to 5 or 6 p.m.) and is considered by most people as unconventional and demanding [1]. The night shift is a special type of shift work that covers at least 3 hours of work between 11 p.m. and 6 a.m. [2]. Shift work is a common practice in modern societies [3] and is present in many professions and occupations to meet customers’ demands [4, 5]. In industrialized societies, approximately 15–20% of workers are employed as shift workers [6]. The report by the National Health Interview Survey and the Occupational Health Supplement estimated that 27% of all workers in the United States (US) in 2015 worked an alternative shift [7]. According to the report, higher rates of alternative shifts were associated with younger age and lower educational levels.

Shift work is associated with adverse health outcomes such as metabolic syndrome, digestive troubles, poor mental health and wellbeing, cancer, and sleep problems [3, 5, 8–10]. Reports by the National Toxicology Program and the International Agency for Research on Cancer concluded that night shift work is probably carcinogenic to humans [11, 12]. Recent literature shows that shift work can affect some aspects of cognitive function, including working memory and attention [13, 14]. Furthermore, a study showed that night Shift workers are at high risk of COVID-19 infection [15] which means that shift workers could be defined as a high-risk group for COVID-infections. The occupational adverse health effects of shift work are one side of the problem. The other side is the potential risk of injury, accidents, and errors due to fatigue, cognitive impairment, and the inability of shift workers to concentrate on performing critical tasks [16–18].

Shift work, especially the night shift, causes circadian misalignment. Circadian misalignment occurs when the inner physiological activities are at a different time than the actual time of the day. Circadian misalignment impairs normal biological processes like insulin sensitivity, immunity, blood pressure, and cardiac autonomic control, leading to different cardiovascular and metabolic health problems [5]. Randomized clinical trials showed that circadian misalignment can cause an increase in blood pressure and inflammatory markers like C- reactive protein, interleukin-6, and tumor necrotic factor, each of which can be an independent risk factor for cardiovascular diseases. To minimize the health risks of shift work, regulations and preventive measures were implemented by governments and manufacturers [19, 20].

In September 2019, the 24th International Symposium on Shiftwork and Working Time (“Shiftwork2019”) was held in the USA by a group of 189 shiftwork and working time professionals [21]. The discussions in the symposium focused on (1) the impacts of shiftwork on work-life balance, sleep, performance, health, and safety across a wider range of domains and (2) on research practice and innovations on shift work to improve the health and safety of shift workers. The adverse health effects of shift work and the risk of occupational accidents and poor performance attracted the attention of scholars from various scientific disciplines. Therefore, a large volume of literature has been published on shift work [3]. The large volume of literature on the topic requires the implementation of a bibliometric analysis of published literature to give scholars and health experts a snapshot of what has been published on the topic, specifically, research hotspots and future research agendas. The conventional bibliometric analysis uses statistical and mathematical tools to analyze scientific research volume, trends, key players, and citation patterns [22]. In modern bibliometric analysis, research topics, network collaborative ties, and the timeline of important topics are mapped and visualized. Bibliometric studies became attractive after the emergence of scientific academic databases such as Scopus and Web of Science since these databases provide researchers and academics with detailed information about scientific publications, including authorship, affiliation, keywords, citations, journals, and year of publication.

Analysis and mapping of published scientific literature on shift work will stimulate policymakers to develop new working schedules that cause the least occupational adverse health effects to employees and maximum benefits to employers. Identifying the volume, the quality of scientific publications, and hot topics on shift work increases the understanding of scholars and the public about the occupational health concerns of shift workers. Bibliometric studies also allow for comparisons between different countries, institutions, or authors. No studies were published to shed light on bibliometric indicators and the mapping of literature on shift work. However, bibliometric studies on circadian rhythm, in general, were published [23, 24].

The current study aimed to give a holistic bibliometric analysis of the scientific literature on shift work across all occupations and professions to identify (1) volume and the annual number of publications produced globally, (2) countries that have produced the most research, (3) journals and authors publishing the most research, (4) extent of research collaboration, (5) most frequent author keywords and terms used in the scientific literature, and (6) the top cited publications that have the greatest scientific impact in the field.

## Method

In the current study, scientific literature on shift work was obtained from Scopus using an extensive list of potential keywords and phrases. Scopus is an academic database, a product of Elsevier, with more than 24 thousand indexed journals in various scientific fields [25]. Scopus provides certain functions that allow for the assessment of research growth and trends. It also allows for the export of data to other programs for the mapping of the literature. Most studies that assessed research growth and patterns, used either Scopus or Web of Science to retrieve the global scientific literature. The fact that Scopus is more inclusive than the Web of Science favors the use of Scopus, which is also available for free to many scholars in low-income countries through the Hinari initiative. A comparative study indicated that about 99.11% of the journals indexed in the Web of Science are also indexed in Scopus [26].

### Search strategy

The keywords used in the research strategy to retrieve relevant documents were shown in Table 1. The search strategy was developed based on review articles related to shift work [5, 27–31]. Different combinations of keywords were used and implemented in the title or title/abstract search. The title/abstract search was implemented using certain restrictions to minimize false-positive results since the title/abstract search might retrieve irrelevant documents. Asterisks and quotation marks were used in writing the keywords to sharpen and widen the search. Different Boolean operators were used in the search query in Scopus. The study period was from January 1st, 2012, to December 31st, 2021. The one-decade period was chosen to allow for better bibliometric analysis and to increase the accuracy of the results since old data might not be relevant and not available in databases [26]. The research strategy included documents published in peer-reviewed scientific journals. Therefore, books and book chapters were not included. Of the retrieved documents, letters, notes, editorials, and errata were excluded.

**Table 1:** Keywords used in the research strategy on shift work using the Scopus database for the period from 2012 – to 2021

Step	strategy	Keywords and limitations	
1	Title search	(title (“night shift* work*” or “night-shift* work*” or (“night job*” and shift*) or “late night work*” or “nightshift work*” or “rotating shift” or “late evening shift*” or “late evening work*” or (“night work*” and shift*) or (“evening work*” and shift) or “rotating shift*” or rotating-shift* or “alternating work*” or alternating-work* or (“non day work*” and shift*) or (“non-day-work*” and shift*) or “shift work*”)) or ((title (night and work* and shift*) or title (night and job and shift*) or title (shift* and work*) or title (shift* and *hour* and work*) or title (*shift*) or title (*work* and sleep* and hour*)) and title-abs (“night shift*” or “shift work*” or “rotating shift*” or “shiftwork*” or “work shift” or “alternating shift”))	5524
2	Title/Abstract search with restrictions	(TITLE-ABS (“night shift*” OR “shift work*” OR “rotating shift*” OR “shiftwork*” OR “shift work” OR “work shift” OR (alternating AND shift) OR “rotating shift”)) AND TITLE (schedul* OR “occupational safety” OR “circadian synchr*” OR “working time arrangement*” OR “work” OR “working” OR shift OR night OR (sleep AND deprivation) OR “circadian disruption” OR “circadian misalignment” OR (circadian AND dysregulation) OR (circadian AND disruption) OR (circadian AND *alignment) OR (circadian AND disturbance*))	7368
2	Inclusion and Exclusion	1. Time: 2012–2021 2. Source type: journal documents only 3. Document type: all types excluding errata 4. Language limitation: None 5. Publication stage: finally published (i.e “article in press” were excluded) 6. Exclusion: experimental studies on animals, cultured cells, or plants	
3	Overall research strategy	Steps (1 OR 2) AND step 3	2328

### Validation of the search strategy

Two colleagues in the field of biomedical sciences volunteered to check the validity of the search strategy. The check for validity consisted of two approaches. In the first approach, the colleagues were asked to confirm the absence of false-positive articles by reviewing 50 articles randomly selected from an Endnote file sent to the reviewers. The research strategy was enhanced by the feedback from the volunteers. In the second approach, the volunteering colleagues were asked to compare the number of publications of the top active authors with the actual number of articles for each scholar by investigating his or her Scopus profile. The results obtained from the two methods were compared by correlation testing to determine significance and the correlation coefficient. This approach was used to confirm the absence of false-negative results. The approach was adopted from previously published bibliometric studies [32].

### Data export and data analysis

The documents obtained from the research strategy were exported to the Microsoft Excel program. The exported information included:

1. general characteristics of the retrieved documents, including the type of documents, encountered languages, and type of access.
2. subject areas of the journals publishing the retrieved articles.
3. most frequent author keywords presented in a network visualization map. In the network visualization map of author keyword co-occurrences, items are presented as nodes, and the larger the node size, the higher the frequency of the item in the retrieved documents. The distance between items represents relatedness.
4. annual growth of publications. Data on the annual number of publications were used to calculate the annual growth rate (AGR), average annual growth rate (AAGR), compound annual growth rate (CAGR), and doubling time (DT). The annual growth rate (AGR), defined as the percentage change in the number of publications for one year, was calculated based on the following equation:

$$AGR={\left[\left( Ending\ Value\hbox{-} Beginning\ Value\right)/ Beginning\ Value\right]}^{\ast }\ 100$$

The average annual growth rate (AAGR) is the average change in the value of a measurement over the study period. The compound annual growth rate (CAGR) provides a constant rate of return over the study period [33]. Its formula is as follows:

$$CAGR=\left[{\left( Ending\kern0.17em Value/ BeginningValue\right)}^{\left({}^{1}\!\left/ \!{}_{n-1}\right.\right)}\right]-1$$

where “n” is the number of years.

The growth analysis was also presented as the “relative growth rate” (RGR), which was defined as the increase in the number of publications per unit of time. The RGR was calculated based on the following equation [34]:

$$RGR=\left[{\mathit{\log}}_e\ {W}_2\hbox{-} {\mathit{\log}}_e\ {W}_1\ \right]/\left(T 2\hbox{-} T 1\right)$$

where log_e_ W_1_: log of the initial number of articles;

*log*_*e*_ *W*_*2*_ : log of the final number of articles after a specific period of interval;

and T2-T1: the unit difference between the initial and final times.

The RGR can be presented in a different format called “doubling time” (DT), defined as the time required for the number of publications to double in number in one year and was calculated based on the following equation [34]:

$$DT= 0.693/ RGR$$

(5) Top 10 active countries are presented in a table.
(6) Cross-country research collaboration of countries with a minimum contribution of 10 publications presented as a network visualization map using the free online VOSviewer program [35]. The node size represents the relative number of collaborating countries while the thickness of connecting lines represents the strength of collaboration (i.e., joint research publications).
(7) The top 10 active authors and author-author degree of collaboration. Prolific authors and author-author degree of collaboration. Author details were exported from Scopus to Microsoft Excel, where authorship analysis was carried out. Analysis in Microsoft Excel included the number of single-authored, two-authored, three-authored, and multi-authored (joint) articles. Analysis of overall collaboration in the field was calculated using the following equation [36]:

$$\textrm{Degree}\ \textrm{of}\ \textrm{collaboration}=C={N}_m/{N}_m+{N}_s$$

where *N*_*m*_ = number of multi-authored papers and *N*_*s*_ = number of single-authored papers.

(8) The top 10 active institutions are presented in a table.
(9) The top 10 active journals are presented in a table.
(10) Citation analysis presented as the Hirsch index (h-index), and top-cited articles.

## Results

### General characteristics of the retrieved articles

Between 2012 and 2021, 2328 documents related to shift work were published in peer-reviewed scientific journals. Of these, 1888 (81.1%) were research articles, 217 (9.3%) review articles, 76 (3.3%) letters, 72 (3.1%) notes, 35 (1.5%), 28 (1.2%), and 12 (0.5%) short surveys. More than one-third of the retrieved articles (*n* = 1095, 47.0%) were available from open-access sources.

The retrieved articles were published in 22 different languages, mainly English (*n* = 2100, 90.2%). The remaining documents were written in non-English but have bilingual abstracts (English and non-English). The presence of bilingual abstracts is a condition imposed by Scopus on all non-English journals that are indexed in Scopus database. The most common non-English languages encountered in the retrieved documents were German (*n* = 45, 1.9%), followed by French (*n* = 34, 1.5%), Russian (*n* = 34, 1.5%), Spanish (*n* = 26, 1.1%), The dominance of English language is partially because the majority of Scopus-indexed journals are English journals with fewer percentage of non-English journals.

### Subject areas of the retrieved articles

Shift work is of concern to several scientific fields. The Scopus database has categorized the retrieved articles into 26 subject areas. Table 2 presents the top 10 subject areas on shift work research. The “medicine” subject area has the highest number of publications (*n* = 1559, 70.0%), followed by nursing (*n* = 315, 13.5%), biochemistry/molecular biology/genetics (*n* = 293, and social sciences (*n* = 245, 10.5%). Because certain journals may be categorized in more than one field, there was an overlap in the subject areas of “shift work” research and the total number was greater than the retrieved number of articles.

**Table 2:** Top 10 subject areas of documents on shiftwork (2012–2021)

Rank	Subject area	Number of publications	% (***N*** = 2328)^a^
1	*Medicine*	1559	67.0
2	*Nursing*	315	13.5
3	*Biochemistry, Genetics and Molecular Biology*	293	12.6
4	*Social Sciences*	241	10.4
5	*Engineering*	165	7.1
6	*Neuroscience*	153	6.6
7	*Environmental Science*	137	5.9
8	*Psychology*	104	4.5
9	*Multidisciplinary*	67	2.9
10	*Business, Management and Accounting*	59	2.5
10	*Health Professions*	59	2.5

^a^Total exceeds 100% because of overlap between different subject areas

### Keyword co-occurrence analysis

**Fig. 1:**
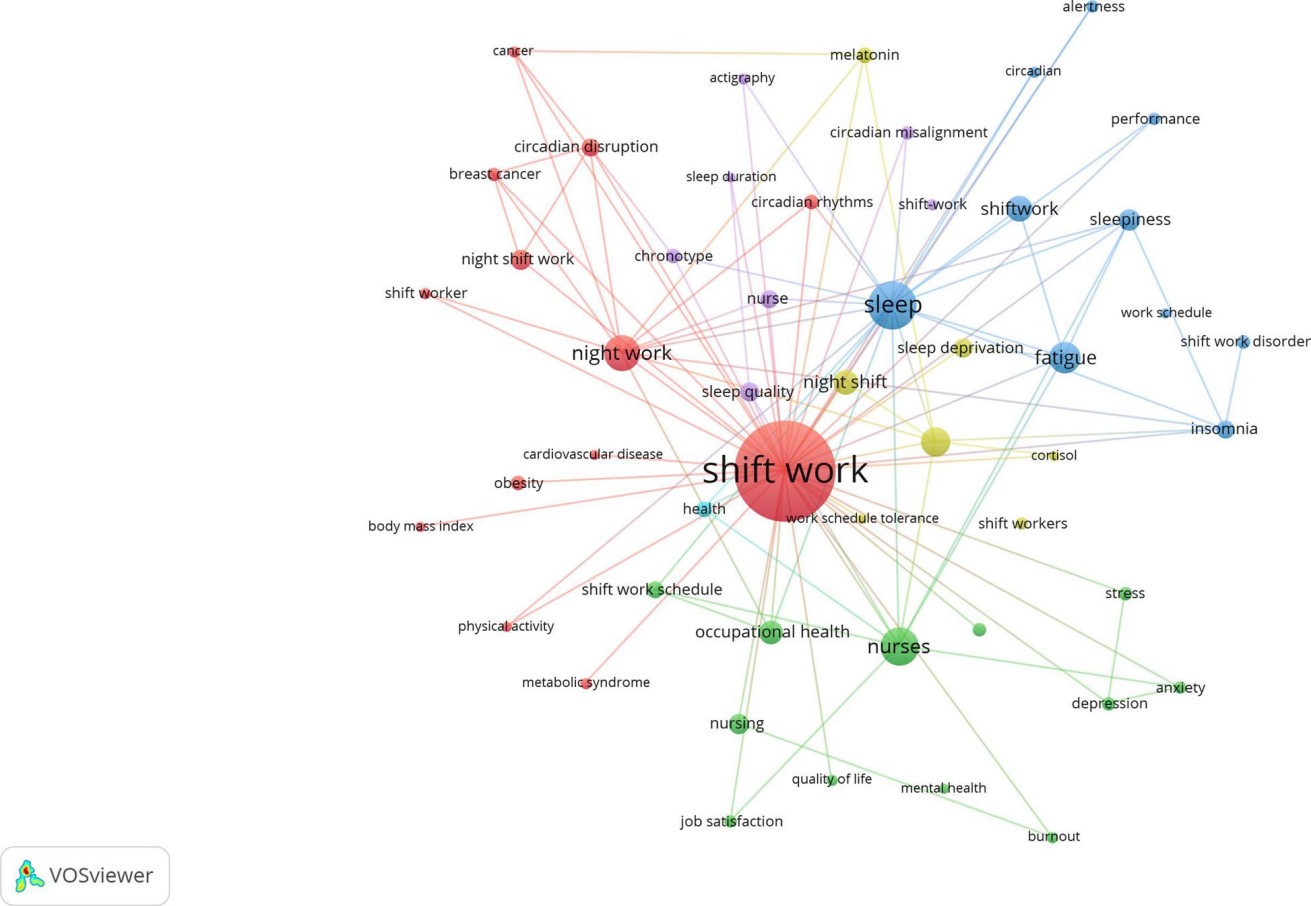
Network visualization map of most frequently occurring (*n* = 5) author keywords in the retrieved documents on shift work (2012–2021). The node size is proportional to the frequency of occurrence while the distance between keywords reflects relatedness

Keyword co-occurrence analysis was carried out to identify research hotspots and future research directions of the academic field. In this study, a keyword co-occurrence visualization map was created by VOSviewer program. The top 50 author keywords were mapped. The most frequent author keywords in the dataset, excluding keywords related to shift- and night work, were sleep, nursing, fatigue, circadian rhythm, and circadian disruption (Fig. 1)

### Growth of publications and citations

**Fig. 2:**
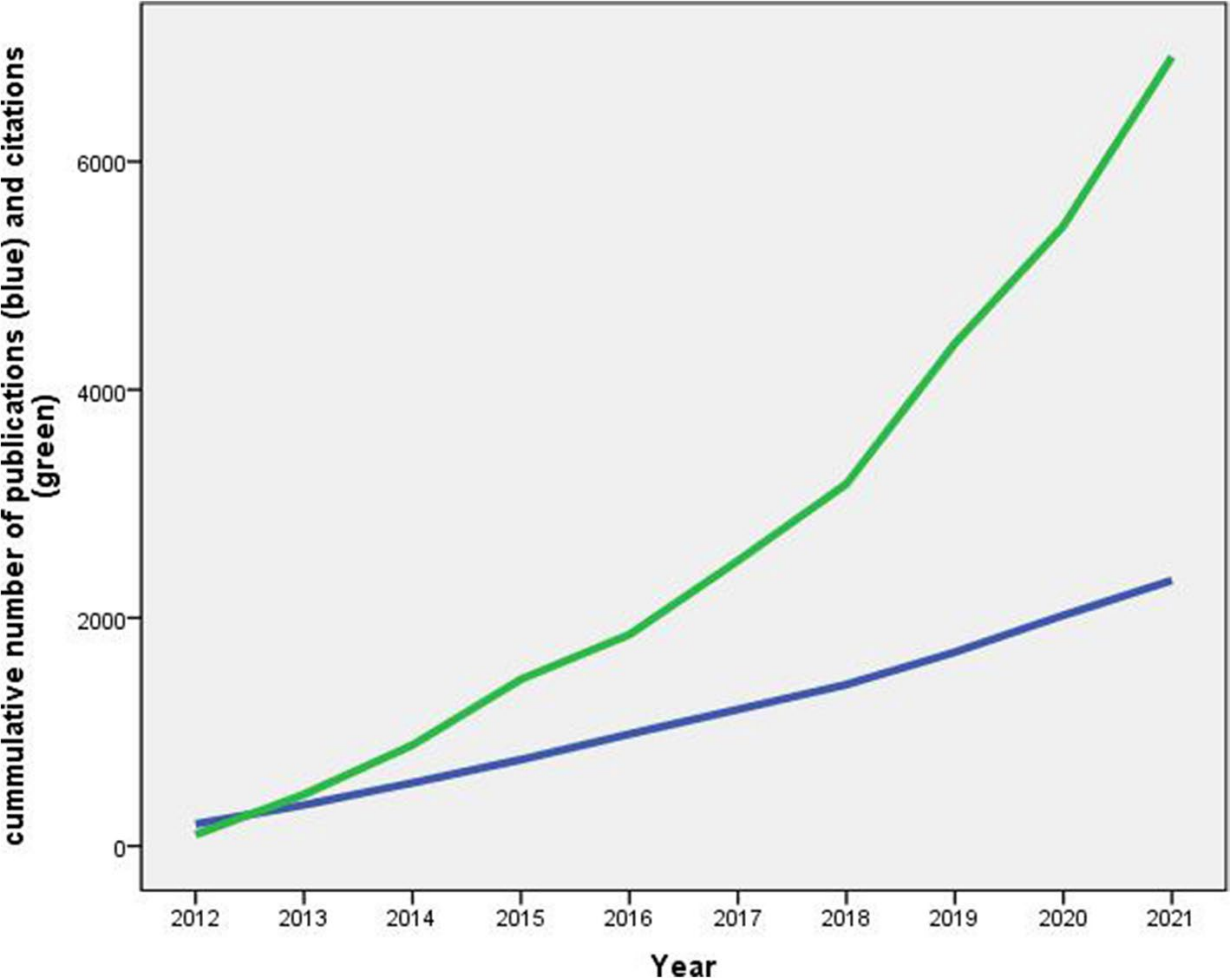
Cumulative number of publications and citations on shift work (2012–2021)

The publication trend in the field is demonstrated in Table 3. The mean number of publications during the study period was approximately 233 documents per year. The highest number of published documents was 323 (13.9%) achieved in 2020. Growth analysis indicated that the highest AGR during the study period was 23.9%. The AGR had three negative values, indicating certain fluctuations during the study period. The RGR declined from 0.6 in 2013 to 0.2 in 2021. The DT increased from 1.2 in 2012 to 3.5 in 2021. Figure 2 presents the cumulative number of publications and the cumulative number of citations per year. The graph shows that the cumulative number of publications follows a linear pattern, indicating a constant rate of new publications during the study period (approximately 200 publications per year). On the other hand, the cumulative number of citations shows an exponential pattern, indicating a dramatic increase in the number of citations toward the end of the study period.

**Table 3:** Annual number of publications, AGR, CAGR, RGRT, and DT on shift work (2012–2021)

Year	Frequency	% ***N*** = 2328	AGR	CAGR	Cumulative total publications	Log_**e**_ ***W***	RGR	DT
2012	193	8.3	–	–	193	5.3	–	–
2013	167	7.2	- 13.5	−15.6	360	5.9	0.6	1.2
2014	194	8.3	16.2	13.9	554	6.3	0.4	1.7
2015	203	8.7	4.6	4.4	757	6.6	0.3	2.3
2016	225	9.7	10.8	9.8	982	6.9	0.3	2.3
2017	216	9.3	- 4.0	- 4.2	1198	7.1	0.2	3.5
2018	217	9.3	0.5	0.5	1415	7.3	0.2	3.5
2019	285	12.2	31.3	23.9	1700	7.4	0.1	6.9
2020	323	13.9	13.3	11.8	2023	7.6	0.2	3.5
2021	305	13.1	- 6.2	−5.9	2328	7.8	0.2	3.5
			AAGR = 5.9	ACAGR = 4.3			Mean RGR = 0.3	Mean DT = 3.2

*AGR* Annual Growth Rate, *AAGR* Average Annual Growth Rate, *RGR* Relative Growth Rate, *DT* Doubling Time, *CAGR* Compound Annual Growth Rate, *ACGAR* Average Compound Annual Growth Rate

### Spatial distribution of publications

The retrieved articles were published by authors from 87 different countries/territories. The country with the most publications was the US (*n* = 504, 21.6%), followed by Australia (*n* = 178, 7.6%), and the UK (*n* = 140, 6.0%). Table 4 lists the top 10 active countries (core countries) in the field. The list included four English-speaking countries. Publications by authors from Canada received the highest number of citations (*n* = 22.9 citations per document) followed by those from the US (*n* = 22.4) and Australia (*n* = 18.4). No significant correlation (Pearson correlation test) was found (*p* = 0.058) between the number of publications for each country and the nominal GDP per capita of the investigated countries. When the number of publications was standardized by GDP (nominal per capita) per capita, Iran had the highest productivity (*n* = 49.2) followed by Brazil (*n* = 16.6) and China (9.1) (Table 5).

**Table 4:** Top 10 active countries publishing on shift work (2012–2021)

Rank	Country	Number of publications	% (***N*** = 2328)	Total Citations	Number of citations per document	^a^GDP (nominal) per capita (*10^**3**^)	Number of publications per GDP (nominal)/ 1000 capita
1	United States	504	21.6	11280	22.4	63.4	7.9
2	Australia	178	7.6	3271	18.4	51.7	3.4
3	United Kingdom	140	6.0	2323	17.0	41.1	3.4
4	Germany	133	5.7	1981	15	46.2	2.9
5	Italy	125	5.4	1628	13.0	31.7	3.9
6	Iran	118	5.1	931	7.9	2.4	49.2
7	Brazil	113	4.9	1405	12.4	6.8	16.6
8	Canada	110	4.7	2522	22.9	43.3	2.5
9	South Korea	101	4.3	1085	10.7	31.6	3.2
10	China	95	4.1	1404	14.8	10.4	9.1

^a^*GDP* Gross Domestic Product (GDP) was Obtained from the World Bank data (2021) [70]

**Table 5:** Top 10 active countries publishing on shift work (2012–2021) standardized by GDP (nominal) per capita

Country	Number of publications	% (N = 2328)	Total Citations	Number of citations per document	^a^GDP (nominal) per capita (*10^**3**^)	Number of publications per GDP (nominal)/ 1000 capita
United States	504	21.6	11280	22.4	63.4	7.9
Australia	178	7.6	3271	18.4	51.7	3.4
United Kingdom	140	6.0	2323	17.0	41.1	3.4
Germany	133	5.7	1981	15	46.2	2.9
Italy	125	5.4	1628	13.0	31.7	3.9
Iran	118	5.1	931	7.9	2.4	49.2
Brazil	113	4.9	1405	12.4	6.8	16.6
Canada	110	4.7	2522	22.9	43.3	2.5
South Korea	101	4.3	1085	10.7	31.6	3.2
China	95	4.1	1404	14.8	10.4	9.1

^a^Obtained from the World Bank data (2021) [37]

### Visualization of cross-country research collaboration

**Fig. 3:**
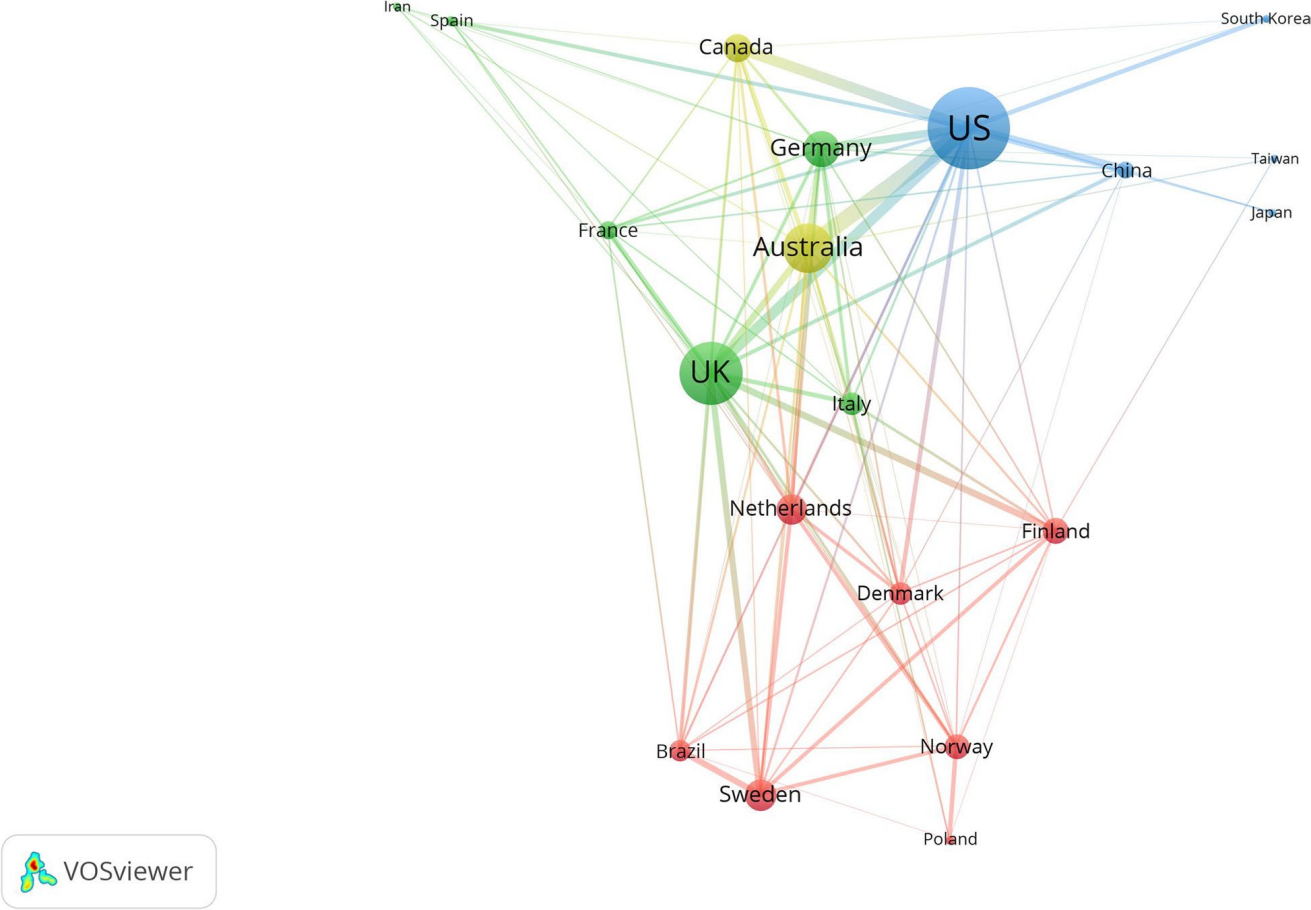
Network visualization map of cross-country (international) research collaboration in the field of shift work (2012–2021). Countries with minimum contribution of 50 publications were included (*n* = 20). Countries with the large node size have the highest number of publications with international authors. The thickness of the connecting line denotes strength of research collaboration

Figure 3 shows the network visualization map for cross-country (international) research collaboration among countries with a minimum contribution of 50 documents each. The map included 20 countries. Countries on the map with the largest node size had the highest number of documents with international authors and were located in the center of the map. On the other hand, countries located at the periphery of the map (e.g. Iran, Taiwan, South Korea, Japan, and Poland) had the least number of documents with international authors. Countries with thick connecting lines had a high number of joint publications. The connecting line between the US and Australia was the thickest, indicating the presence of relatively high numbers of joint publications between the two countries.

### Authorship analysis, author-author degree of collaboration, and prolific authors

In total, 10,516 author names appeared on the retrieved documents, giving an average of 4.5 author names per document. There were 290 (12.5%) single-authored publications. The number of multi-authored publications was mainly those with five or more authors (*n* = 731, 31.4%). Table 6 shows the authorship pattern and author-author degree of collaboration. The degree of collaboration among authors was 87.5%. The degree of author-author collaboration showed an increasing pattern, with approximately 84% during the early times of the study period and reaching approximately 93% toward the end of the study period. Table 7 shows the top 10 active authors. Bjorvatn, B. (Norway) was the most prolific author with 36 (1.5%) publications, followed by Pallesen, S. (Norway) with 33 (1.4%) publications. The list of active authors included four researchers from Norway, two from Finland, and two from the US.

**Table 6:** Authorship pattern on shift work (2012–2021)

Number of authors	Frequency	% (***N*** = 2383)
One	290	12.5
Two	298	12.8
Three	353	15.2
Four	352	15.1
Five	301	12.9
More than five	731	31.4
Degree of collaboration	2038/290 *100 = 87.5%

**Table 7:** Top 10 active authors publishing on shift work (2012–2021)

Rank^a^	Author Name	Number of publications	% (***N*** = 2328)	Country affiliation
1	Bjorvatn, B.	36	1.5	Norway
2	Pallesen, S.	33	1.4	Norway
3	Dorrian, J.	28	1.2	Australia
4	Harma, M.	24	1.0	Finland
5	Puttonen, S.	21	0.9	Finland
5	Vetter, C.	21	0.9	US
7	Garde, A.H.	20	0.9	Denmark
7	Moen, B.E.	20	0.9	Norway
9	Banks, S.	19	0.8	Australia
9	Schernhammer, E.S.	19	0.8	US
9	Waage, S.	19	0.8	Norway

^a^In the ranking system, two equal institutions were given the same rank and one position is skipped

**Fig. 4:**
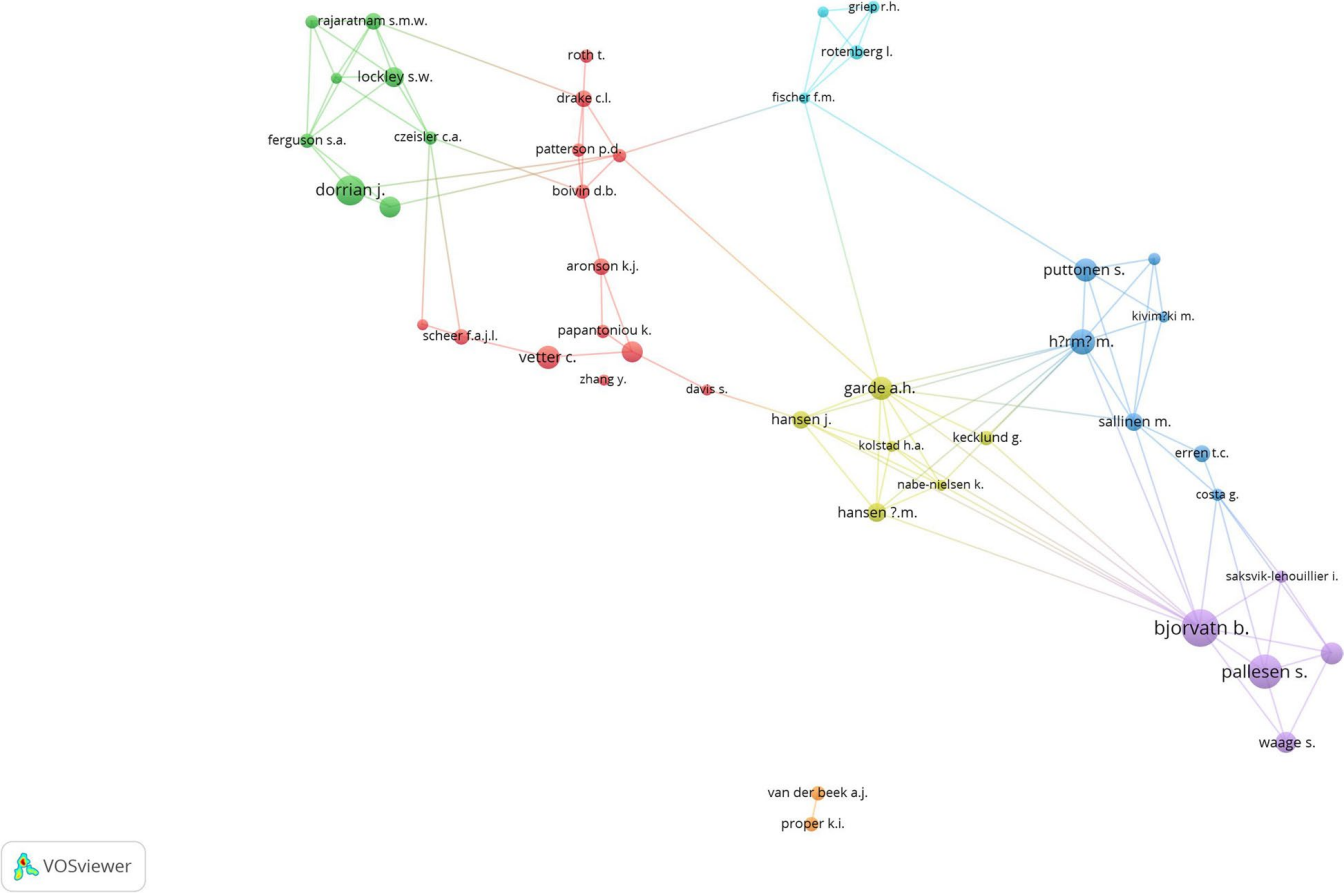
Network visualization map of author-author degree of collaboration in the field of shift work (2012–2021). Authors with minimum contribution of 10 publications were included (*n* = 51). Only authors who existed in a research network were shown (*n* = 45). Authors with similar color constituted a collaborative research network

Figure 4 is a network visualization of collaborative ties among authors who published at least 10 documents in the dataset (*n* = 51). Fifty-one authors met the criteria. However, six of them did not fit into any research group and therefore were not shown on the map. The map shows 45 authors distributed into seven clusters, five of them included five or more researchers contributing to the development of the field. The largest collaboration network represents a research group composed of 13 scholars affiliated with institutions in the US and Canada.

### Top ten active institutions/organizations in shift work research

Table 8 lists the top 10 institutions/organizations on “shift work” research. أHarvard University (*n* = 97, 4.2%) was the most prolific institution in the field followed by Brigham and Women’s Hospital (U.S) (*n* = 86, 3.7%), and the Finnish Institute Of Occupational Health (Työterveyslaitos) (*n* = 43, 1.8%) and the University of Bergen (*n* = 43, 1.8%).

**Table 8:** Top 10 institutions/organizations publishing articles on shift work (2012–2021)

Rank^a^	Institutions/Organization	Number of publications	% ***N*** = 2328	Country Affiliation
1	*Harvard University*	97	4.2	US
2	*Brigham and Women’s Hospital*	86	3.7	US
3	*Työterveyslaitos (Finnish Institute of Occupational Health)*	43	1.8	Finland
3	*University of Bergen*	43	1.8	Norway
5	*Haukeland University Hospital*	38	1.6	Norway
6	*Monash University*	37	1.6	Australia
7	*University of South Australia*	35	1.5	Australia
8	*University of Copenhagen*	33	1.4	Denmark
9	*Universidade de Sao Paulo*	32	1.4	Brazil
10	*Stockholm University*	30	1.3	Sweden

^a^In the ranking system, two equal institutions were given the same rank and one position is skipped

### Top 10 active journals in publishing documents on shift work

The retrieved articles were disseminated through 991 scientific journals. Table 9 lists the top 10 active journals in publishing documents on shift work. The *Chronobiology International* journal (publisher: Taylor & Francis) ranked first with 103 (4.4%) documents, followed by the *Occupational and Environmental Medicine* journal (publisher: BMJ Publishing Group) (*n* = 59, 2.5%) and *International Journal of Environmental Research and Public Health* (publisher: MDPI) (*n* = 47; 2.0%).

**Table 9:** Top 10 active journals publishing documents on shift work (2012–2021)

Rank	Journal title	Number of publications	% N-2328	CiteScore^a^	Publisher
1	*Chronobiology International*	103	4.4	4.4	Taylor & Francis
2	*Occupational and Environmental Medicine*	59	2.5	6.8	BMJ Publishing Group
3	*International Journal of Environmental Research and Public Health*	47	2.0	3.4	MDPI
4	*Scandinavian Journal of Work Environment and Health*	40	1.7	6.8	Finnish Institute of Occupational Health
5	*International Archives of Occupational and Environmental Health*	34	1.5	4.0	Springer Nature
6	*Plos One*	32	1.4	5.3	Public Library of Science
7	*Industrial Health*	30	1.3	3.3	National Institute of Industrial Health
8	*Journal of Occupational and Environmental Medicine*	28	1.2	2.7	Wolters Kluwer Health
9	*Applied Ergonomics*	23	1.0	7.1	Elsevier
9	*Scientific Reports*	23	1.0	1.0	Nature Publishing Group
9	*Sleep*	23	1.0	8.0	Oxford University Press
9	*Work*	23	1.0	2.0	IOS Press

^a^CiteScore: strength parameter developed by Elsevier. The value of CiteScore for each journal was obtained from Scimajo journal and country rank

### Citation analysis

The retrieved documents received 32,301 citations with an H-index of 71. Table 10 shows the top 10 cited documents in the field of shift work [3, 38–45]. Six articles were review articles. The top-cited articles investigated the impact of shift work on health, including diabetes mellitus and cancer. One of the top-cited documents investigated the impact of shift work on nursing job satisfaction and burnout.

**Table 10:** Top ten cited articles on shift work (1944–2021)

Rank	Title	Year	Source title	Cited by	Type
1	“Health consequences of shift work and insufficient sleep”	2016	BMJ (Online)	459	Review
1	“Circadian misalignment augments markers of insulin resistance and inflammation, independently of sleep loss”	2014	Diabetes	360	Article
3	“The association between long working hours and health: A systematic review of epidemiological evidence”	2014	Scandinavian Journal of Work, Environment and Health	338	Review
4	“Shift work and cancer risk: Potential mechanistic roles of circadian disruption, light at night, and sleep deprivation”	2013	Sleep Medicine Reviews	293	Review
5	“Negative impacts of shiftwork and long work hours”	2014	Rehabilitation Nursing	289	Article
6	“Shift work and diabetes mellitus: A meta-analysis of observational studies”	2015	Occupational and Environmental Medicine	276	Review
7	“Impacts of shift work on sleep and circadian rhythms”	2014	Pathologie Biologie	245	Review
8	“Endogenous circadian system and circadian misalignment impact glucose tolerance via separate mechanisms in humans”	2015	Proceedings of the National Academy of Sciences of the United States of America	236	Article
9	“Shift work and the assessment and management of shift work disorder (SWD)”	2013	Sleep Medicine Reviews	234	Review
10	“The longer the shifts for hospital nurses, the higher the levels of burnout and patient dissatisfaction”	2012	Health Affairs	229	Article

## Discussion

The increasing prevalence of shift work across most professions throughout the world and the adverse health and social effects of shift work led to the accumulation of a large volume of scientific literature on the topic. In the current study, the author analyzed and assessed the scientific literature on shift work to give an overview of the evolution, growth, and key players in the field.

Most of the retrieved documents were published in journals categorized in the subject area of “medicine”. However, there was a good share of publications in the “nursing” subject area. Most nurses worldwide work night shifts to cover patient care across 24 hours, leading to health and social problems for nurses and risks of poor performance and errors [5, 16, 46, 47]. Nurses opt to work the night shifts for various financial and family reasons, but they are mostly unaware of the chronic negative health implications of working night shifts [48–50]. Shift work negatively affects the quality of life of shift workers, especially women, due to insufficient time for marital and child care [51, 52]. Shift work negatively affects the individual’s mental health, including psychological distress, anxiety, and depression [31, 53, 54].

The current study showed a positive value for AAGR. This increase could be due to (1) an increase in the prevalence of shift work across different societies and professions, (2) the natural increase in the number of scholars and global research productivity in general, (3) the emergence of many specialized journals in the field of occupational health and sleep medicine, and (4) the appearance of studies linking shift work to serious health consequences such as cancer and cardiometabolic disorders.

Keyword analysis and the top-cited articles indicated that various negative health consequences, sleep deprivation, fatigue, nursing, and circadian disruption were major hot topics in the field. Shift work is associated with short- and long-term health problems [55]. For example, insomnia, fatigue, and sleep disturbances are related to the acute effects of shift work while potential cardiometabolic and cancer health problems are related to the chronic effects of shift work [3, 9, 10, 53, 56, 57]. A systematic review suggested that the increased health risks in shift workers may be due to the desynchronization of the circadian rhythm, that alters the normal regulation of physiological functions in the body [58].

Journals in the core list were mainly in the field of occupational health and sleep medicine. Journals in the field of nursing or mental health were under-represented. Shift workers constitute a good percentage in developed countries. For example, in Europe, 21% of the workforce is engaged in some type of shift work [59]. This relatively large proportion of the population requires special attention, and further research is needed to raise awareness and develop safer working schedules for workers across professions with 24-hour working systems. The presence of several occupational/work health journals and leading sleep journals emphasizes that shift work, especially, the night shift is unhealthy and may be dangerous.

The countries in the active list were mainly high-income and industrialized countries, including the US, Australia, and the UK. This is not surprising since high-income countries have well-established infrastructure and resources for scientific research as evident in previously published bibliometric studies [60, 61]. Australia ranked second in this field despite its poor contribution to the field as measured one decade ago [62]. One potential reason for this finding is the increased health and medical research investment in Australia which led to a noticeable increase in PubMed publications from Australia [63]. A bibliometric analysis of the top 100 cited articles on sleep medicine found that two-thirds of the articles were published by authors in the US followed by Canada, the UK, Germany, and Australia [64]. Another bibliometric study of the top 100 cited chronotype research papers showed that Australia was in the sixth rank at the global level [24]. These studies showed that Australian researchers have active role in research related to sleep, biological clock, and circadian rhythm, which might explain their rank on the shift work research.

Certain developing countries such as Iran, China, and Brazil were also present in the core list. The emergence of these developing countries in the top active list is due to rapid industrialization and the need for shift working schedules in these countries. Furthermore, the increasing number of Scopus-indexed journals published by institutions and research centers in developing countries increased the visibility of research output from these countries. There has been a noticeable increase in the number of journals in the fields of public and occupational health, as well as sleep medicine, from various countries and world regions in the past two decades. Currently there are more than 100 health related journals published from Iran and indexed in Scopus.

In 1982, a study by Mahathevani, R. indicated that in developing countries, such as Malaysia and Singapore, there was a total lack of documentation on the different shift systems and that scientific literature on the effects and consequences of shift work was absent [65]. The author of the study as well as consequent studies indicated that shift work was heavily influenced by gender, social factors, type of industry, cultural values, and religious norms of the countries [66]. The limited contribution of certain countries and regions is mostly due to limited research resources and capacities rather than the absence of the problem. In a survey study of 1400 shift workers across 20 countries and all industries, 90% of the shift workers feel that they contribute to the success of their organization, 50% consider themselves to be essential workers, and 69% feel concerned about their job security [67].

The core countries included Canada, which ranked 8th on the list. However, publications from Canada were highly impactful. Cohort studies on the cost and health injuries due to shift work in Canada drew the attention of researchers and policy workers across the world. A cohort study conducted in Canada on 30,000 Canadians between the ages of 16 to 65, showed that from 1996 to 2006, the number of worker injuries decreased by 27.9% while the rate of injury among night shift workers remained stable [68]. From 1996 to 2006, the Canadian Labour force increased by 21.7%, with almost half of this growth in non-regular shift work. In 2006, there were 2.7 million lost time injury compensation claims awarded in Canada with approximately 107,000 claims among men and 200,000 claims from women, secondary to the high risk of injury associated with shift work. A study on the estimate of shift work indicated that in 2011, there were 1.8 million (12% of the working population) Canadians exposed to the night shiftwork [69].

Author-author collaboration and cross-country collaboration maps indicated the presence of noticeable collaboration networks at the author level but not at the cross-country level. Most of the collaboration networks were between researchers in countries that share cultural, geographical, or linguistic factors. This explains the existence of countries such as Iran and Poland at the periphery of the map with a limited number of links with other countries. The opposite applies to countries such as the US, the UK, Canada, and Australia. The inadequate international research collaboration in the field of shift work between researchers in developed and developing countries is a barrier to the development of this field. Shift work is a global phenomenon, and international collaboration among different countries is needed to strengthen research in this field and increase the visibility of shift worker-related work and health problems.

### Strength and limitations

Several bibliometric studies were published to analyze and map the literature on sleep, circadian disruption, melatonin, and chronotype research [23, 24, 64, 70]. However, there was one scientometric article published on shift work in 2011 in the German language [62]. The main strengths of the current study were (1) the comprehensiveness of the research strategy without language or subject restrictions and (2) the comprehensiveness of the analysis of the retrieved data, such that the current study can be considered a future reference for researchers in the field of shift work. However, the current study has a few limitations. The use of Scopus is a point of strength and a point of weakness. Scopus is a large academic database of all fields. However, the use of a single database makes the results less than perfect. The research strategy, despite validation, is not without error. Therefore, potential false-positive and negative results remain a possibility that needs to be considered by readers. The use of title search is expected to minimize any research errors.

## Conclusions and recommendations

In the current study, scientific literature on shift- and night work was retrieved, analyzed, and interpreted using the bibliometric approach to explore the content, countries, institutions, authors, and journals involved in the field. The analysis revealed steep annual growth in publications over the last decade, with US authors and institutions dominating. Content analysis indicated the impact of shift work on sleep quality, risk of cardiometabolic diseases, and cancer. Of the professions most investigated by the retrieved literature was nursing. The current study is the first to conduct a bibliometric analysis of the literature on shift work, and it identified several hot topics that merit further investigation and research. Policymakers can build on the current study to implement human resources management that promotes better working shift schedules and a safer work environment.

## Data Availability

All data presented in this manuscript are available on the Scopus database using the search query listed in the methodology section.

## Abbreviation

WHO: World Health Organization
